# Recurrent vs. Nonrecurrent Superficial Non-Healing Corneal Ulcers in Cats: A Multifactorial Retrospective Analysis

**DOI:** 10.3390/ani15142104

**Published:** 2025-07-16

**Authors:** Nuanwan Rujirekasuwan, Panpicha Sattasathuchana, Natthanet Sritrakoon, Naris Thengchaisri

**Affiliations:** 1 Ophthalmology Unit, Faculty of Veterinary Medicine, Kasetsart University Veterinary Teaching Hospital, Kasetsart University, Bangkok 10900, Thailand; nuanwan.ru@ku.th (N.R.); fvetnnsk@ku.ac.th (N.S.); 2 Department of Companion Animal Clinical Sciences, Faculty of Veterinary Medicine, Kasetsart University, Bangkok 10900, Thailand; fvetpcs@ku.ac.th

**Keywords:** cat, cornea, eye, indolent ulcer, refractory corneal ulcer, surgery

## Abstract

Feline superficial non-healing corneal ulcers are persistent lesions requiring individualized treatment to reduce recurrence. This retrospective study evaluated 136 affected eyes (113 nonrecurrent; 23 recurrent) to identify clinical and treatment-related factors associated with recurrence. Recurrent ulcers were more common in older cats (7.2 ± 4.3 vs. 5.1 ± 4.6 years; *p* = 0.026). Domestic Shorthairs were the most frequently affected breed (50%), and central ulcer location predominated in both groups. Recurrent cases required more intensive management, with 16.9% needing ≥ 2 treatment courses, compared to 83% of nonrecurrent cases resolving after a single course. Healing time following corneal debridement was longer in recurrent cases (32.3 ± 34.4 vs. 25.5 ± 23.1 days; *p* = 0.272), and corneal sequestrum occurred more frequently (13.0% vs. 10.6%; *p* = 0.735). Corneal debridement was the primary treatment modality. Systemic medications were more often used in recurrent cases, notably oral lysine (47.8% vs. 26.5%; *p* = 0.049) and famciclovir (17.4% vs. 2.6%; *p* = 0.016). Recurrent cases also showed significantly higher rates of concurrent viral (*p* < 0.001) and bacterial/fungal infections (*p* = 0.027). In conclusion, recurrent superficial non-healing corneal ulcers were associated with age and systemic illness, emphasizing the need for early diagnosis and management of underlying conditions.

## Simple Summary

This study evaluated 136 feline eyes with superficial non-healing ulcers, comparing outcomes between recurrent (113 eyes) and nonrecurrent cases (23 eyes) based on treatment timing, methods, medications, and complications such as age, breed, sex, and other factors. Recurrent corneal ulcers occurred significantly more commonly in older cats and were more frequently seen in females and non-brachycephalic breeds. Recurrent ulcers were more likely to be bilateral, required longer and more intensive treatment, and had higher rates of corneal sequestrum. Treatment included corneal debridement, topical and systemic medications, and surgery for complicated cases. Recurrent cases were more frequently associated with receiving systemic medications, including lysine and famciclovir. Concurrent systemic diseases, especially infections, were more common in the recurrent group, suggesting underlying illness contributes to recurrence. These ulcers are more frequent in older, systemically ill cats and require longer treatment. Early diagnosis and addressing both ocular and systemic health support better recovery.

## 1. Introduction

Corneal ulcers are among the most frequently encountered ophthalmic disorders in veterinary practice and require appropriate diagnosis and management to prevent complications and preserve vision [1]. A superficial non-healing corneal ulcer, also referred to as an indolent corneal ulcer, refractory ulcer, or persistent corneal erosion, is characterized by delayed epithelial healing and chronic corneal epithelial defects in cats. These ulcers typically appear as superficial erosions but persist due to a failure of the corneal epithelium to adhere properly to the underlying stroma. Impaired epithelial regeneration, abnormal basement membrane formation, or defective epithelial–stromal adhesion are believed to play a role in their pathogenesis [2,3,4]. While similar conditions in dogs have been well documented under various terms, including indolent ulcers, Boxer ulcers, and spontaneous chronic corneal epithelial defects, corresponding cases in cats remain less extensively studied. In cats, these ulcers are often associated with middle-to-older age [5,6] or underlying ocular conditions such as keratoconjunctivitis sicca, eyelid malformations, lagophthalmos, trauma, or feline herpesvirus type 1 (FHV-1) infection [5,7]. Other predisposing factors include chronic corneal trauma, breed predisposition, neurologic deficits, and iatrogenic injury following procedures such as grid keratotomy, which may also increase the risk of corneal sequestrum formation [6].

Treatment approaches depend on ulcer severity and range from conservative medical therapy to surgical intervention. Corneal debridement, either with dry cotton-tipped applicators or a diamond burr, is a common first-line intervention aimed at removing loose epithelium and promoting the adhesion of healthy epithelial cells [1,6,8,9,10]. Adjunctive therapies such as serum eye drops, topical growth factors, polysulfated glycosaminoglycans, substance P, insulin-like growth factor-1 (IGF-1), and prophylactic topical antibiotics have been employed to enhance epithelial healing and prevent infection [3,11,12]. Systemic anti-inflammatory medications may also be indicated to manage ocular inflammation [13]. In more severe or refractory cases, surgical management may be required, including superficial keratectomy, conjunctival graft placement, or the use of bandage contact lenses [5,7,14,15]. Despite the availability of various treatment modalities, superficial non-healing corneal ulcers in cats remain a clinical challenge, and data on treatment outcomes are limited. A better understanding of the factors that influence healing and recurrence is critical for guiding clinical decision-making.

The aim of this study was to investigate various clinical features and treatment-related aspects of feline superficial non-healing corneal ulcers by comparing recurrent and nonrecurrent cases, focusing on the healing outcomes such as healing time, incidence of corneal sequestrum, and onset timing after treatment, and assess the relationship between different surgical interventions and recurrence. Additionally, this study also examined the association between specific medications and ulcer recurrence, as well as comparing the incidence of concurrent diseases between the two groups.

## 2. Materials and Methods

### 2.1. Study Design and Ethical Approval

This retrospective study was conducted at the Ophthalmology Unit, Kasetsart University Veterinary Teaching Hospital, Faculty of Veterinary Medicine, Kasetsart University, Bangkok, Thailand. Medical records were reviewed for feline patients presented between March 2019 and November 2024. Ethical approval was obtained from the Kasetsart University Institutional Animal Care and Use Committee (approval number ACKU68-VET-026) and from the Ethical Review Board of the Office of the National Research Council of Thailand (NRCT license U1-07457-2561).

### 2.2. Case Selection and Data Collection

A total of 121 client-owned cats diagnosed with superficial non-healing corneal ulcers without stromal involvement were included in the study. Cases were identified using the hospital’s electronic medical records system. Information retrieved from each case included patient signalment (age, sex, and breed), affected eye, duration of clinical signs, history of ocular trauma, prior medications, complications, healing time, and recurrence.

### 2.3. Classification of Cases

Of the 121 cats evaluated, 101 cats (113 eyes) were classified as having nonrecurrent superficial non-healing corneal ulcers. The mean age of cats in this group was 5.1 years (SD 4.6; range: 0.2–19.6 years). The breeds represented included forty-five Domestic Shorthairs (44.6%), twenty-five Persians (24.8%), ten British Shorthairs (9.9%), nine Exotic Shorthairs (8.9%), four American Wirehairs (4.0%), four Maine Coons (4.0%), two Scottish Folds (2.0%), one Himalayan (1.0%), and one Bengal (1.0%).

Recurrent superficial non-healing corneal ulcers were identified in 20 cats (23 eyes), with all recurrences affecting the same eye as the initial episode. For the recurrent group, data from the first clinical presentation were included in the analysis. The healing duration was recorded for each treatment course until complete resolution. The group comprised eight males and twelve females, with a mean age of 7.2 years (SD 4.9; range: 0.5–18.7 years). Breeds in the recurrent group included ten Domestic Shorthairs (50.0%), five Persians (25.0%), two Ragdolls (10.0%), and one each of British Shorthair, Exotic Shorthair, and Scottish Fold (5.0% each). A summary of the breed distribution and demographic characteristics of each group is provided in Table 1.

### 2.4. Ophthalmic Examination and Treatment Procedure

**Figure 1:**
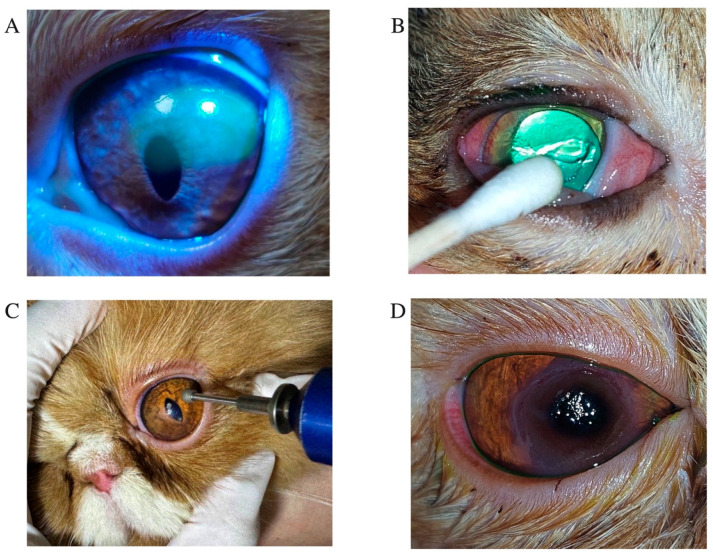
Clinical presentation, treatment approaches, and sequelae observed in feline superficial non-healing corneal ulcers. (**A**) A feline non-healing corneal ulcer is characterized by a superficial corneal ulcer with persistent, chronic corneal epithelial defects. (**B**) A Domestic Shorthair cat with a superficial non-healing corneal ulcer undergoing corneal debridement treatment using cotton-tipped applicators. (**C**) Application of a diamond burr in a circular motion over the ulcerated area to remove damaged corneal tissue. (**D**) Appearance of corneal sequestrum in a Persian cat with a chronic non-healing corneal ulcer.

Ophthalmic examinations were performed to confirm the presence of superficial non-healing corneal ulcers in all enrolled cats (Figure 1). All examinations were conducted by a board-certified veterinary ophthalmologist. Clinical evaluation included slit-lamp biomicroscopy using a portable slit lamp (SL-15, Kowa, Tokyo, Japan) to assess corneal abnormalities. Superficial non-healing corneal ulcers were identified using commercially available fluorescein paper ophthalmic strips (32 K. Supply Co., Ltd., Bangkok, Thailand), based on the presence of a persistent corneal epithelial defects with positive fluorescein uptake and the detection of a surrounding rim of loosely adherent or non-adherent epithelial cells (epithelial lip) (Figure 1A). The location of each corneal lesion was described using standard anatomical references (central, lateral, medial, dorsal, or ventral) and recorded.

Various concurrent diseases were identified based on clinician assessments and medical records. Viral infections were broadly categorized as feline herpesvirus-1 (FHV-1) or feline upper respiratory tract infections with concurrent ocular signs (e.g., conjunctivitis, dendritic corneal lesions, or keratitis suggestive of FHV-1), as well as feline infectious peritonitis (FIP) presenting with uveitis. Bacterial and fungal infections included perineal wound infections, pyoderma, otitis externa with aural hematoma, dermatophytosis caused by *Microsporum canis*, and bilateral purulent otitis externa. Endocrine and metabolic disorders included diabetes mellitus and systemic hypertension, which were associated with hyphema and uveitis. Neoplastic conditions included multicentric lymphoma, mediastinal lymphoma, an oral mass (dermatophytic pseudomycetoma), and a retrobulbar mass (squamous cell carcinoma). Neurological disorders involved incomplete or absent palpebral reflexes and facial nerve trauma following total ear canal ablation, as well as traumatic brain injury.

**Figure 2:**
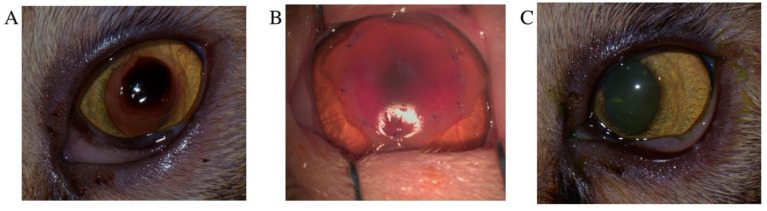
Superficial keratectomy with amniotic membrane transplantation for the treatment of corneal sequestrum. (**A**) Close-up of the preoperative appearance of corneal sequestrum in a Persian cat. (**B**) Immediate postoperative appearance following superficial keratectomy and amniotic membrane transplantation. (**C**) Eight weeks postoperatively, the cornea showed significant graft clarity and resolution of the preexisting corneal vascularization.

Treatment procedures were standardized across cases. One drop of topical anesthetic (0.5% tetracaine hydrochloride ophthalmic solution; Alcon Laboratories, Puurs, Belgium) was applied to the affected eye. Cats were gently restrained using the ‘kitty burrito’ technique, and eyelids were manually held open. Corneal debridement was performed using sterile cotton-tipped applicators in a circular motion (Figure 1B), or with a diamond burr in cases of pronounced epithelial thickening or diffuse non-adherence (Figure 1C). Diamond burr debridement was performed using an Algerbrush II (Alger Equipment Co., Inc., Lago Vista, TX, USA), fitted with a 3.5 mm diamond tip applied in a circular motion for approximately one minute to remove non-adherent epithelium [10]. Following corneal and diamond burr debridement, all patients received topical antibiotic therapy based on clinician preference, including tobramycin 0.3% ophthalmic solution (Tobrex^®^, Alcon-Couvreur, Puurs, Belgium) or moxifloxacin ophthalmic solution (Vigamox^®^, Alcon-Couvreur NV, Puurs, Belgium), administered four times daily, or oxytetracycline hydrochloride with polymyxin B sulfate ointment (Terramycin^®^, PT. Pfizer Indonesia, Jakarta, Indonesia), applied three times daily to the affected eye. Artificial tears including containing 0.1% or 0.3% sodium hyaluronate (Hialid^®^, Santen Pharmaceutical Co., Ltd., Ishikawa, Japan) were administered to the affected eye every 6–8 h. Systemic analgesics were also prescribed at the clinician’s discretion: tolfenamic acid (2–4 mg/kg PO q24h), robenacoxib (1–2 mg/kg PO q24h), or meloxicam (0.1 mg/kg PO initially, then 0.05 mg/kg PO q24h for three days). In recurrent cases suspected to involve feline herpesvirus type 1 (FHV-1), oral lysine (Optixcare^®^, Burlington, ON, Canada; 500 mg/cat q24h) and systemic famciclovir (FAMVIR^®^, Novartis Pharma, Milano, Italy; 30–60 mg/kg PO q12h) were administered, with dosage adjusted based on lesion severity. In cases complicated by corneal sequestrum (Figure 1D), superficial keratectomy combined with amniotic membrane transplantation was performed under general anesthesia using an ophthalmic surgical microscope (ZEISS OPMI Lumera^®^ I, Carl Zeiss Meditec AG, Jena, Germany) [16,17]. Representative images are shown in Figure 2. Amniotic membranes were obtained from human placentas via elective cesarean section, processed under sterile conditions (Thai Red Cross Eye Bank, Bangkok, Thailand). Sequestrum excision was performed using a microsurgical blade, followed by placement of the amniotic membrane over the defect without tension. The membrane was sutured in a simple interrupted pattern using 9-0 monofilament polyglycolic acid (PGA; FSSB Chirurgische Nadeln GMBH, Jestetten, Germany). Temporary tarsorrhaphy was maintained for two weeks.

Postoperative care included the application of an Elizabethan collar, along with topical and systemic administration of broad-spectrum antibiotics. Moxifloxacin ophthalmic solution and artificial tears containing 0.1% or 0.3% sodium hyaluronate were administered to the affected eye every 6 h. Systemic antibiotic therapy consisted of oral amoxicillin–clavulanate (20 mg/kg PO q12h). Nonsteroidal anti-inflammatory drugs (NSAIDs) were continued to control postoperative inflammation. Cases were classified as healed when no fluorescein stain uptake was observed (Figure 2C). Healing time was defined as the interval from initial treatment to the first negative fluorescein stain. Postsurgical healing time was defined as the duration from surgery to complete suture dissolution and discontinuation of medical therapy.

### 2.5. Statistical Analysis

Statistical analyses were performed using GraphPad Prism version 9 (GraphPad Software, Boston, MA, USA) and IBM SPSS Statistics version 22 (IBM Corporation, Chicago, IL, USA). Sample size calculations were conducted to ensure 80% power at a significance level (α) of 0.05. Continuous data were tested for normality using the Shapiro–Wilk test. Descriptive statistics were used to summarize clinical characteristics. Comparisons between recurrent and nonrecurrent superficial non-healing corneal ulcers were made using unpaired *t*-tests for normally distributed continuous variables. Categorical variables, including surgical interventions, medication use, and concurrent diseases, were analyzed using Fisher’s exact test or binomial distribution tests as appropriate. A *p*-value of <0.05 was considered statistically significant.

## 3. Results

A total of 136 eyes from 121 cats were included in this study. Nonrecurrent superficial non-healing corneal ulcers accounted for 113 eyes (83.1%), while recurrent cases comprised 23 eyes (16.9%). Most cases in both groups were unilateral, with bilateral involvement in 11.8% of nonrecurrent and 15% of recurrent cases. Cats with recurrent corneal ulcers were significantly older (mean age 7.2 years; SD 4.3; range 0.5–18.7 years) compared to those with nonrecurrent corneal ulcers (mean age 5.1 years; SD 4.6 years; range 0.2–19.6 years; *p* = 0.026).

Recurrent corneal ulcers were more frequently observed in female and non-brachycephalic breeds. Domestic Shorthairs were the most commonly affected breed in both groups. There were no statistically significant differences in sex, breed, body weight, healing time by treatment type, or incidence of corneal sequestrum between the nonrecurrent and recurrent groups (*p* > 0.05 for all; Table 1 and Table 2). The total number of treatments required in cats with non-healing corneal ulcers varied between groups. The majority of cats in the nonrecurrent group (83%) responded to a single course of treatment. In contrast, 14.7% of recurrent cases required two treatment courses, and 2.2% required three or more. Healing time varied depending on the treatment modality, with similar patterns observed across groups. Mean healing time following corneal debridement was slightly longer in recurrent cases compared to nonrecurrent ones (32.3 ± 34.4 days; range 7–133 days vs. 25.5 ± 23.1 days; range 6–161 days), although the difference was not statistically significant. Healing durations following diamond burr debridement or corneal debridement with surgery were also comparable between groups (Table 2).

Corneal sequestrum was identified as a complication in 15 cats: 12 cases (10.6%) in the nonrecurrent group and 3 cases (13.0%) in the recurrent group. In nonrecurrent cases, sequestra developed in 10 cats (8.8%) after corneal debridement (mean onset 17.6 ± 14.8 days) and in 2 cats (1.8%) after diamond burr debridement (mean onset 13.0 ± 7.0 days). Among recurrent cases, the mean time to sequestrum development was 19.3 ± 15.0 days. One recurrent case with sequestrum resolved spontaneously without surgery after 133 days. No significant differences were observed between groups regarding the incidence or timing of sequestrum development (Table 2).

The majority of superficial non-healing corneal ulcers were centrally located in both groups, accounting for 89.4% (101/113 eyes) of nonrecurrent and 87.0% (20/23 eyes) of recurrent cases. Lateral lesions were more frequently observed in recurrent cases, at 13.0% (3/23 eyes), compared to nonrecurrent cases, at 7.9% (9/113 eyes). Medial, dorsal, and ventral corneal ulcers were rare and occurred exclusively in nonrecurrent cases, each representing 0.9% (1/113 eyes) of the group. Overall, central corneal ulcers comprised 89.0% of all lesions, followed by lateral. at 8.8%, and less than 1% for each of the remaining positions. There was no statistically significant association between corneal ulcer location and recurrence (*p* = 0.804).

Comparing the incidence of cats treated for superficial non-healing corneal ulcers based on duration before eye examination in recurrent and nonrecurrent cases, most cats in both groups were examined within one week of corneal ulcer onset, including 60 cases (53.1%) in the nonrecurrent group and 12 cases (52.2%) in the recurrent group. A total of 25 nonrecurrent (22.1%) and three recurrent (13.0%) cases presented between 2–4 weeks, with a higher proportion in the nonrecurrent group. Delayed presentation beyond four weeks occurred in 28 nonrecurrent (24.8%) and eight recurrent (34.8%) cases. No statistically significant differences were observed between groups regarding the time from corneal ulcer onset to initial ophthalmic examination (*p* > 0.05 for all comparisons).

Corneal debridement was the most commonly performed procedure in both groups, accounting for 88 cases (77.9%) in the nonrecurrent group and 20 cases (87.0%) in the recurrent group (Table 2). Diamond burr debridement was used in 12 nonrecurrent cases (10.6%) and in one recurrent case (4.3%). A combination of debridement and surgery was performed in 13 nonrecurrent cases (11.5%) and two recurrent cases (8.7%). No statistically significant differences were observed between groups in the distribution of treatment modalities (*p* > 0.05 for all comparisons; Table 3).

Medications for superficial non-healing corneal ulcers included the use of artificial tear drops (sodium hyaluronate), which were administered in the majority of cases in both groups, with no significant difference between nonrecurrent (93.8%) and recurrent (95.6%) cases (*p* = 1.000). Autologous serum eye drops were used infrequently and only in recurrent cases, without significant difference between groups (Table 4).

The use of oral NSAIDs was comparable between groups (40.7% in nonrecurrent vs. 30.4% in recurrent cases; *p* = 0.351). Oral lysine supplementation was prescribed significantly more often in recurrent cases (47.8%) compared to nonrecurrent cases (26.5%; *p* = 0.049). Similarly, oral famciclovir was used more frequently in recurrent cases (17.4%) than in nonrecurrent cases (2.6%; *p* = 0.016). Oral antibiotics, including amoxicillin-clavulanate and doxycycline, did not significantly differ between groups (*p* = 0.285; Table 4).

Among the concurrent diseases, viral infections were significantly more prevalent in the recurrent group (95% CI: 11.6–49.2%) compared to the nonrecurrent group (95% CI: 1.8–10.6%; *p* < 0.001). Bacterial or fungal infections (such as dermatitis or otitis externa) were also significantly associated with recurrence (*p* = 0.027). No statistically significant differences were observed between groups for other concurrent conditions, including renal disease, endocrine/metabolic disorders, neoplasia, neurological disease, gastrointestinal/hepatic disease, or trauma (*p* > 0.05; Table 5). Conversely, the absence of concurrent diseases was more common in nonrecurrent cases (95% CI: 65.3–81.6%) than in recurrent cases (95% CI: 8.1–44.0%; *p* < 0.001) (Table 5).

## 4. Discussion

The present retrospective study investigated the prevalence, treatment, and outcomes of non-healing corneal ulcers in cats, focusing on recurrent versus nonrecurrent cases. In the present study, nonrecurrent superficial corneal ulcers were more common, with both groups typically occurring in older cats and in Domestic Shorthair and Persian breeds, supporting previous reports of increased susceptibility in these populations [2,5,6]. Cats with recurrent corneal ulcers (typically occurring in mature-aged cats) were significantly older than those with nonrecurrent cases (typically occurring in adult-aged cats), with mean ages of 7.2 ± 4.3 years and 5.1 ± 4.6 years, respectively. No association was found between recurrence and corneal ulcer location, time to presentation, or treatment modality, suggesting that recurrence may be influenced more by patient-related factors than by clinical management. Notably, concurrent diseases were more frequently identified in recurrent cases of superficial non-healing corneal ulcers compared to nonrecurrent cases. These recurrent cases commonly involved FHV-1, ocular respiratory infections (e.g., conjunctivitis, corneal lesions, keratitis), uveitis-linked FIP, and bacterial or fungal skin infections. These findings support the hypothesis that systemic or localized illnesses may impair corneal healing and contribute to ulcer recurrence. Overall, concurrent diseases were significantly more prevalent in recurrent cases than in nonrecurrent cases. Specifically, concurrent viral (*p* < 0.001) and bacterial or fungal infections (*p* = 0.027) were significantly more common in recurrent cases compared to nonrecurrent cases.

Although superficial non-healing corneal ulcers were predominantly located centrally in both recurrent and nonrecurrent cases, as previously reported [6,18], no significant association was identified between corneal ulcer location and recurrence. This suggests that factors such as underlying systemic disease or breed-related anatomical characteristics may have a greater influence on recurrence risk. Bilateral corneal abnormalities were observed in 11.8% of cats with nonrecurrent corneal ulcers and 15% of cats with recurrent ulcers, predominantly in brachycephalic breeds. This may be linked to disease transmission to the contralateral eye or co-infection with FHV-1 [19]. Brachycephalic breeds may be predisposed to corneal disease due to reduced tear production, eyelid abnormalities, and decreased corneal sensitivity [20], which can delay healing and lead to ulceration, keratitis, or sequestrum formation [21]. In dogs, brachycephaly has been previously associated with corneal protrusion and a wider palpebral fissure, leading to a greater risk of exposure keratopathy and trauma-induced ulcers [22]. Furthermore, decreased corneal sensitivity has been shown to impair the blink reflex, compounding ocular surface vulnerability [23,24]. Although limited, current data suggest that brachycephalic feline breeds may share similar anatomical and physiological risk factors. Moreover, tear film abnormalities and reduced tear production are recognized as potential contributing factors in the development and recurrence of corneal ulcers in feline patients [25]. In particular, stressful conditions may suppress tear production, further compromising ocular surface health [26]. Future studies may benefit from incorporating both tear film break-up time (TFBUT) and Schirmer tear testing to better evaluate tear film quality and tear production in cats.

Corneal debridement was the most commonly employed treatment for superficial non-healing corneal ulcers in cats, and no significant difference in healing outcomes was observed between recurrent and nonrecurrent cases. The mean healing time following corneal debridement was 25.46 ± 23.10 days (range: 6–161 days) in nonrecurrent cases and 32.3 ± 34.35 days (range: 7–133 days) in recurrent cases. These results align with prior studies reporting a mean healing time of approximately 30 days (ranging from 7–240 days) for corneal ulcers treated with debridement [6]. For corneal ulcers treated with corneal diamond burr debridement, the mean healing time was 47.5 ± 27.60 days (range: 7–84 days) in nonrecurrent cases and 14 days in recurrent cases, consistent with earlier research reporting a median healing time of 14 days (ranging from 6–39 days) [10].

Superficial keratectomy, known for its high success rate and relatively rapid recovery, has been shown to achieve complete healing within about 14 days [6]. In this study, the healing time after keratectomy with an amniotic membrane was 19.13 ± 13.13 days (range: 15–60 days) for nonrecurrent cases and 29.5 ± 19.09 days (range: 16–43 days) for recurrent cases. Previous reports have estimated similar healing times after surgery with amniotic membrane transplantation of between 28–45 days [27]. The use of an amniotic membrane offers notable benefits, such as promoting healing, minimizing corneal scarring, and improving transparency [16], with no reported recurrences of sequestrum [17]. Despite its effectiveness, superficial keratectomy is not typically the first-line treatment due to factors such as the need for general anesthesia, high procedural costs, and the requirement for advanced microsurgical skill [18]. After corneal debridement or superficial keratectomy, topical antibiotics should be administered four times daily to prevent secondary bacterial infections. In cases of poor tear film quality, topical mucinomimetic agents, such as methylcellulose, hyaluronic acid, and high molecular weight hyaluronan, are recommended and applied four to six times daily [18,28].

The pathogenesis of corneal sequestrum remains unclear, though it is often associated with chronic corneal irritation from entropion, trichiasis, ulcerative keratitis, trauma, and tear film disorders, including FHV-1 infection [7,28,29,30]. In the present study, corneal sequestrum was identified in 10.6% of nonrecurrent and 13.0% of recurrent non-healing corneal ulcers, with a higher predisposition observed in brachycephalic breeds, particularly Exotic Shorthair and Persian cats, followed by Domestic Shorthair, consistent with previous reports [6]. Surgical excision via superficial to deep keratectomy is the mainstay of treatment, often combined with grafting techniques such as conjunctival pedicle grafts, corneoconjunctival transposition, or amniotic membrane transplantation [7,17,29]. Alternative grafts such as porcine small intestinal submucosa, urinary bladder matrix, or bovine pericardium have also been described [28,29,30,31]. Recurrence rates of corneal sequestrum appear lowest with amniotic membrane (0%; 0/7), followed by conjunctival pedicle grafts (11.5%; 6/52) and corneoconjunctival transposition (14.3%; 1/7) [7,17,28,32]. The incidence following corneal debridement in this study (8.8%) closely aligned with prior reports (10%) [6], while sequestrum formation after diamond burr debridement remained low (1.8%), comparable to earlier studies (4.8%) [6]. Notably, previous reports indicated a markedly higher incidence (31%) following grid keratotomy [6].

The increased use of oral lysine and famciclovir in recurrent cases suggests a viral component, particularly FHV-1, in the pathogenesis of non-healing corneal ulcers [33]. While lysine may help reduce the frequency of viral flare-ups by inhibiting replication, its efficacy remains controversial and should be used as part of a broader treatment strategy [34,35]. FHV-1 shedding has been reported in up to 50% of healthy cats, with higher rates of recurrence in multi-cat environments [33]. Oral famciclovir (40–90 mg/kg) has been shown to reduce conjunctivitis and ocular discomfort and promote reepithelialization in cats with FHV-1–associated ocular disease [36,37,38]. Systemic conditions such as carcinoma, feline infectious peritonitis, and diabetes mellitus are known to impair immune function and contribute to ocular surface disorders, such as stress-related autonomic responses, viral infections, reduced tear production, and abnormalities in tear film quality, may predispose cats to ocular surface disease and corneal ulceration [5,7,39,40,41]. Previous studies have reported that 17.6% of cases experienced recurrent non-healing corneal ulcers and delayed healing due to underlying systemic illness [10]. Prolonged administration of corticosteroids in cats with these conditions can worsen corneal ulcers, increase viral shedding, and result in complications such as stromal keratitis and corneal sequestrum [34,42].

**Figure 3:**
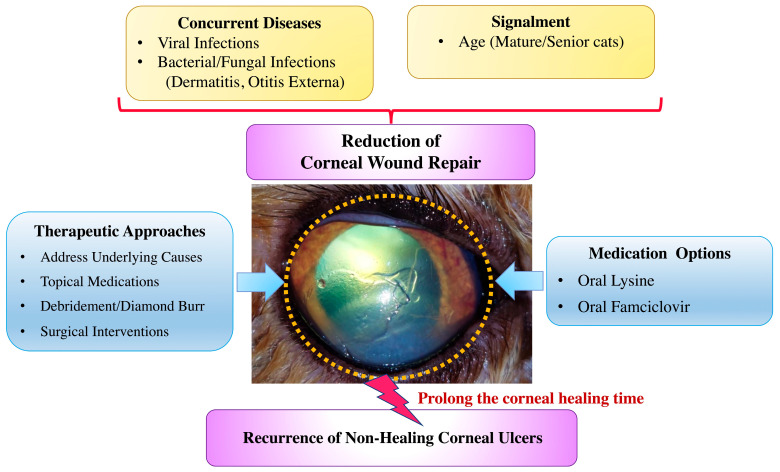
Comparative overview of clinical characteristics, concurrent diseases, and managements in cats with recurrent superficial non-healing corneal ulcers. The present study identifies various potential factors contributing to ulcer recurrence. Management strategies employed in the present study are also summarized.

This study had several limitations, primarily related to its retrospective design, which relied on preexisting medical records and was subject to potential bias, such as incomplete data and inconsistent follow-up. These factors limited the ability to establish causal relationships. The absence of a control group further restricted the identification of risk factors influencing corneal ulcer recurrence and healing outcomes. Although systemic diseases were documented, potential underlying causes such as FHV-1 infection or environmental influences were not thoroughly investigated, and PCR testing was not performed in all cases. The lack of longitudinal data limited the assessment of long-term outcomes and recurrence. A breed bias toward Domestic Shorthair and brachycephalic cats may reduce generalizability, and the small sample size limited statistical power. A comparative overview of patient characteristics, concurrent conditions, and interventions in cats with recurrent superficial non-healing corneal ulcers is presented in Figure 3. The recurrence of feline superficial non-healing corneal ulcers is believed to be influenced by both intrinsic and extrinsic factors, resulting in impaired corneal epithelial proliferation, reduced stromal keratocyte migration, and prolonged inflammation. The promotion of corneal healing involves a multifactorial approach targeting epithelial regeneration and stromal remodeling. Initial management includes mechanical debridement or superficial keratectomy to remove devitalized epithelium, followed by the application of bioactive tear substitutes (e.g., autologous serum or hyaluronic acid) and systemic NSAIDs to modulate inflammation and support re-epithelialization, alongside the management of systemic illnesses. Adjunctive therapies, including platelet-rich plasma, topical growth hormone treatments, and exogenous stem cells, facilitate corneal healing by promoting epithelial proliferation and remodeling of the extracellular matrix. Nonetheless, novel therapeutics are needed to optimize this process by enhancing epithelial stratification, increasing the synthesis of key matrix components (type IV and V collagen and leucine-rich proteoglycan), inhibiting myofibroblast proliferation, and preventing neovascularization to achieve scarless corneal healing.

## 5. Conclusions

In this study, the majority of feline superficial non-healing corneal ulcers were nonrecurrent, while a smaller portion were recurrent. Compared to nonrecurrent cases, recurrent corneal ulcers were more likely to be bilateral and were associated with significantly older cats, typically in the mature age group, whereas nonrecurrent cases tended to occur in adult-aged cats. Domestic Shorthair cats were most frequently affected, followed by Persian cats, which exhibited a relatively high risk of corneal ulcer recurrence. Recurrent corneal ulcer cases required more treatment courses and had longer healing times compared to nonrecurrent cases. Corneal sequestrum occurred in both groups at similar rates, with no significant difference in incidence or timing. Most sequestra were associated with corneal debridement or diamond burr procedures. Importantly, recurrent cases had a significantly higher prevalence of concurrent diseases, especially viral and bacterial/fungal infections such as dermatitis and otitis externa, highlighting a strong association between these comorbidities and ulcer recurrence. Thus, early diagnosis and identification of concurrent systemic diseases during initial treatment of cats with superficial non-healing corneal ulcers are crucial for providing better care and preventing recurrence by managing both ocular lesions and underlying systemic conditions to improve long-term outcomes.

## Figures and Tables

**Table 1:** Clinical characteristics of feline superficial non-healing corneal ulcers in recurrent and nonrecurrent cases.

Parameters	Nonrecurrent Non-Healing Corneal Ulcers	Recurrent Non-Healing Corneal Ulcers
No. of patient	101 (83.5%)	20 (16.5%)
No. of eyes	113 (83.1%)	23 (16.9%)
Lesion in 1 eye	89 (88.1%)	17 (85.0%)
Lesion in 2 eyes	12 (11.8%)	3 (15.0%)
Age (years)	5.1 ± 4.6	7.2 ± 4.3 *
Gender		
Male	57 (56.4%)	8 (40.0%)
Female	44 (43.5%)	12 (60.0%)
Non-brachycephalic cat	55 (54.5%)	12 (60.0%)
Brachycephalic cat	46 (45.5%)	8 (40.0%)
Breed		
Domestic Shorthair	45 (44.6%)	10 (50.0%)
Persian	25 (24.8%)	5 (25.0%)
British Shorthair	10 (9.9%)	1 (5.0%)
Exotic Shorthair	9 (8.9%)	1(5.0%)
American Wirehair	4 (4.0%)	-
Maine Coon	4 (4.0%)	-
Scottish Fold	2 (2.0%)	1 (5.0%)
Himalayan	1 (1.0%)	-
Bengal	1 (1.0%)	-
Ragdoll	-	2 (10.0%)
Body weight (kg)	4.20 ± 1.54	4.77 ± 1.50

Note: * *p* < 0.05 vs. the nonrecurrent group.

**Table 2:** Comparison of healing time, corneal sequestrum incidence, and onset timing after treatment of feline superficial non-healing corneal ulcers in recurrent (*N* = 23) and nonrecurrent (*N* = 113) cases.

Parameters	Nonrecurrent Non-Healing Corneal Ulcers	Recurrent Non-Healing Corneal Ulcers	*p*-Value
Healing time (days)			
Debridement	25.5 ± 23.1 (*N* = 88)	32.3 ± 34.4 (*N* = 20)	0.272
Diamond burr	47.5 ± 27.6 (*N* = 12)	14 (*N* = 1)	-
Debridement + Surgery	50.5 ± 27.6 (*N* = 13)	54.5 ± 8.5 (*N* = 2)	0.694
Healing time after surgery (days)	19.13 ± 13.13 (*N* = 13)	29.5 ± 19.09 (*N* = 2)	0.580
Number of cats with corneal sequestrum (95%CI)	12 (5.94–16.30%)	3 (−0.72–26.81%)	0.735
Exotic Shorthair	5	-	-
Persian	3	-	
Domestic Shorthair	2	2	-
American Wirehair	1 (2 eyes)	-	-
Ragdoll	-	1	-
Day found sequestrum after debridement	17.6 ± 14.8 (*N* = 10, 8.8%)	19.3 ± 15.0 (*N* = 3, 13.04%)	0.871
Day found sequestrum after diamond burr	13.0 ± 7.0 (*N* = 2, 1.77%)	-	-

**Table 3:** Comparison of the incidence of cats treated with different surgical interventions for feline non-healing corneal ulcers in recurrent (*N* = 23) and nonrecurrent (*N* = 113) cases.

Treatment Methods	Nonrecurrent Non-Healing Corneal Ulcers *N* (95% CI)	Recurrent Non-Healing Corneal Ulcers *N* (95% CI)	*p*-Value
Debridement	88 (70.2–85.5%)	20 (73.2–100.0%)	0.326
Diamond burr	12 (4.9–16.3%)	1 (−4.0–12.7%)	0.351
Debridement + Surgery	13 (5.6–17.4%)	2 (−2.8–20.2%)	0.695

**Table 4:** Association of several medications with feline superficial non-healing corneal ulcers in recurrent (*N* = 23) and nonrecurrent (*N* = 113) cases.

Medications	Nonrecurrent Non-Healing Corneal Ulcers, *N* (%)	Recurrent Non-Healing Corneal Ulcers, *N* (%)	*p*-Value
Artificial tear drops			
Yes	106 (93.8%)	22 (95.6%)	1.000
No	7 (6.2%)	1 (4.4%)	
Autologous serum eye drops			
Yes	2 (1.8%)	0 (0%)	1.000
No	111 (98.2%)	23 (100.0%)	
Oral NSAIDs			
Yes	46 (40.7%)	7 (30.4%)	0.351
No	67 (59.3%)	16 (69.6%)	
Oral lysine			
Yes	30 (26.5%)	11 (47.8%)	0.049
No	83 (73.5%)	12 (52.2%)	
Oral famciclovir			
Yes	3 (2.6%)	4 (17.4%)	0.016
No	110 (97.4%)	19 (82.6%)	
Oral antibiotics			
Amoxicillin-clavulanate	6 (5.3%)	0 (0%)	0.285
Doxycycline	43 (38.1%)	8 (34.8%)	
No antibiotics	64(56.6%)	15 (65.2%)	

**Table 5:** Comparison of the incidence of concurrent diseases in cats with recurrent (*N* = 23) and nonrecurrent (*N* = 113) superficial non-healing corneal ulcers.

Concurrent Disease	Nonrecurrent Non-Healing Corneal Ulcers *N* (95% CI)	Recurrent Non-Healing Corneal Ulcers *N* (95% CI)	*p*-Value
Viral infection	7 (1.8–10.6%)	7 (11.6–49.2%) **	<0.001
Renal disease	5 (0.6–8.2%)	3 (0.7–26.8%)	0.109
Endocrine/Metabolic disorders	4 (0.1–6.9%)	1 (−4.0–12.7%)	0.851
Neoplasia/Mass	4 (0.1–6.9%)	2 (−2.8–20.2%)	0.272
Bacterial/Fungal infections	3 (−0.3–5.6%)	3 (−0.7–26.8%) *	0.027
Neurological disease	3 (−0.3–5.6%)	1 (−4.0–12.7%)	0.661
Gastrointestinal/Hepatic diseases	2 (−0.7–4.2%)	0	0.520
Trauma	2 (−0.7–4.2%)	0	0.520
Total (with concurrent diseases)	30 (18.4–34.7%)	17 (11.6–49.2%)	0.703
No concurrent disease	83 (65.3–81.6%)	6 (8.1–44.0%) **	<0.001

Note: * *p* < 0.05, ** *p* < 0.01 vs. the nonrecurrent group.

## Data Availability

The data presented in this study are available on request from the corresponding author.

## Abbreviations

The following abbreviations are used in this manuscript:

FHV-1	feline herpesvirus type 1
NSAIDs	nonsteroidal anti-inflammatory drugs
SD	standard deviation
